# Racism, research, and the human condition

**Published:** 2019-07-24

**Authors:** Marcel D’Eon

**Affiliations:** 1 University of Saskatchewan, Saskatchewan, Canada

At a recent workshop I attended, the facilitators asked us all to describe how people look when they are open for a conversation. Many people willingly offered the standard signals: smile, open posture, and, of course, not looking at their phone. As usual, I spoiled the party confessing that I did not know how to tell if someone was open to connecting. I was being radically candid based on my own many mistakes.

Not so long ago, while biking along a path in the west end of Saskatoon, I came up behind three youths walking abreast thus “blocking” the entire path. They were dressed in what I believe was “Goth”: trench coats (it was the middle of the summer), long unkempt hair, piercings and tattoos (I have two of my own), and other dark clothing. I recognized these outfit as likely “Goth” because for a time that was, during his forced high school career, my son’s chosen garb. (He is now entering a Master’s program on a scholarship.)

Not wanting to kindle their ire, I pedaled a few metres back, unobtrusively. Shortly after, one of the youths noticed me. I don’t have the quietest bike. He stepped in front of his friends leaving me a clear opportunity to pass. Knowing that people behave the way they are treated and wanting to keep a calm situation from escalating (fear and ignorance muddles one’s brain), in my calmest and most sincere sounding voice with the hint of a warm smile, as I passed the party of three, said, “Thank you!” The lad who had let me pass - of the three the one whose appearance really shouted, “Rebel” and “I don’t give a s—t” - responded, “You are welcome, **Sir**!” I nearly fell off my bike for surprise at what came out of his mouth. Moreover, I was ashamed at how badly I had misjudged (and thought later how often I may have done so on a regular basis without even realizing it). I was clearly guilty of *clothesism*. The way people present themselves does not accurately reflect the inner person. I foolishly judged those books by their covers.

There have been other recent examples along the same theme where I misjudged … badly. Perhaps, to answer again the question posed at the workshop, “how do you recognize someone open for interaction?” the way people respond may have more to do with how I initially interact with them than their own dispositions. Maybe a warm invitation to them, one that intentionally communicates openness and a willingness to connect, can conjure up a similar response. I do notice (warning – unscientific findings) that when I pass strangers on the sidewalk and am able to meet their furtive glance with a smile and/or a nod of peace sign I am rewarded - almost always - with a smile or similar gesture in return. Perhaps the people I encounter are mirrors exhibiting behaviours in response to what they see in me. We may get what we expect. I feel I cannot predict how people will act by how they look, who they are by their appearance.

To amplify and fix this seemingly simple point into your memory - as it seems to be in mine - I offer this quote from “A Gentleman in Moscow”:^3^

*…, what can a first impression tell us about anyone? Why, no more than a chord can tell us about Beethoven, or a brushstroke about Botticelli. By their very nature, human beings are so capricious, so complex, so delightfully contradictory, that they deserve not only our consideration, but our reconsideration—and our unwavering determination to withhold our opinion until we have engaged with them in every possible setting at every possible hour*.

Making incorrect judgments about people is, at the base, bad research: poor data collection, premature closure, false generalizations, confirmation biases, and other insidious cognitive errors. Racism, too, at the base, consists of at least poor research. Wikipedia tells me that there are two common definitions of racism. The first seems to be the one most often used in the media and at the coffee shop: “prejudice, discrimination, or antagonism directed against someone of a different race *based on the belief that one's own race is superior* (emphasis mine).” This first type of racism I will call malignant racism. The second definition provided by Google is actually the foundation, the real reason why the first type of racism thrives, and is included within the first definition: “*the belief* (emphasis mine, again) that all members of each race possess characteristics or abilities specific to that race, especially so as to distinguish it as inferior or superior to another race or races.” This type of racism I call ignorant, lacking a full understanding, perhaps due to poor research.

To hold the belief that one’s own race is superior, one needs first to believe that there is something such as a race. Then we need to believe that most people of one race are very similar to others in that race, more than they share similarities with people of other races. We need to believe that our differences define us and not that our common humanity shapes us. We also need to believe that there are objective criteria by which one can judge races and individuals within races, and that judging is actually a legitimate activity even if it were accurate. The road to malicious racism (the first kind described by Wikipedia) seems long and tortuous. One has to commit many cognitive and I think moral errors. Once people, often intellectuals and politicians, establish a dominant, dogmatic discourse based on race, the malignant variety of racism takes hold. Once entrenched it is hard to uproot.

Can the truth, obtained through rigorous research, save us from racism? Likely not on its own. We also need the disposition and openness to allow our views to be challenged and modified. And we need, perhaps most importantly, the willingness to connect with others who are different. Among people of good will, courageous research can set us free to embark on a different path. Through open dialogue and friendly argumentation,^1,2^ hearts and minds can change and eventually we can restrain both ignorant and malignant racism.

As you read and respond to the articles in this issue, we hope that you will think creatively and courageously,^4^ that you will learn to expose wrong thinking and poor research and root out the prejudice it feeds.

Rajendran with the image and accompanying text, “The emotional brainbow,” provides us with some profound insights on the beauty and complexity of one person genuinely connecting with another. This is an antidote to racism. We need people who are willing and able to connect with fellow human beings, however unfamiliar.

Dubé et al. in “It takes a community to train a future physician: social support experienced by medical students during a community-engaged longitudinal integrated clerkship” describe, from the perspective of medical students, the social supports that allow them to adapt to and meet the demands and challenges of a longitudinal integrated clerkship.

Harms et al. in “From good to great: learners’ perceptions of the qualities of effective medical teachers and clinical supervisors in psychiatry” explore the excellent educator by examining narrative comments from psychiatry faculty evaluations. They analyzed almost three hundred narrative comments to discover what undergraduate and postgraduate learners notice about effective educators in psychiatry. Researchers have already observed many characteristics before. Novel themes include the importance of relationships, learner security, and inspiration through role modeling.

Smith and her team in “The Calgary student run clinic in context: a mixed-methods case study” explore how stakeholders in the University of Calgary’s student run clinic perceived its purpose. Drawing on 13 interviews and electronic medical records, they found there was great uncertainty about the role of the clinic. Most people saw the clinic as an effective referral provider while others thought of the clinic as primarily an educational unit.

Abbiati et al. in “Construct and predictive validity of the Strength of Motivation for Medical School- Revised (SMMS-R) questionnaire: a French validation study” evaluated the construct and predictive validities of the French version of the Strength of Motivation for Medical School-Revised questionnaire (SMMS-R-FR). Using a sample of 372 students at three French-speaking medical schools in France and Switzerland, they confirmed the three-factor structure of the original SMMS-R questionnaire. Both Total Strength of Motivation and Readiness to Start positively predicted a deep learning approach and negatively predicted a surface learning approach. Willingness to Sacrifice positively predicted a deep learning approach and Persistence negatively predicted a surface learning approach. Educators should use these findings cautiously as predictive power here does not indicate a causal relationship.

Burgess and her team from McMaster University in **“**Lines in the sand: pre-interview rank and probability of receiving admission to medical school**”** explore how the probability of receiving an offer varies based on the applicants’ pre-interview rank. This may help determine whether the cut-off point for number applicants offered interviews is congruent with the probability these applicants will be given an offer of admission. Using 2,659 pre-interview rankings from 2013-2017, they calculated a linear-by-linear association Chi-square test and a Spearman Correlation between pre- and post-interview ranks. They found that all applicants had between a 50.0% and 76.4% chance of admission. These results indicate that the cut off for interviews does not include individuals with a relatively low chance of admission.

Orsino and Ng in **“**Can adaptive expertise, reflective practice, and activity theory help achieve systems- based practice and collective competence?” explore the practice context of autism spectrum disorder through the framings of collective competence and activity theory. They then connect with and argue that adaptive expertise and reflective practice will prepare learners to be more responsive to the dynamic needs of the systems in which they work.

Hunter and Thomson in “A scoping review of social determinants of health curricula in post-graduate medical education” used only studies where program had described, implemented and evaluated such curricula. Academic outcomes were frequently positive. One study reported a positive patient- related outcome. They incorporated these results into design recommendations for a post-graduate curriculum to address social determinants of health.

Monteiro and Xenodemetropoulos in “Resident Practice Audit in Gastroenterology (RPAGE): an innovative approach to trainee evaluation and professional development in medicine” describe the development of RPAGE and its utility. RPAGE captures assessments of knowledge, professionalism, and technical skills, in real time using an electronic platform. Using 2635 anonymized competence assessment data the authors analyzed the acceptability of the audit and tested the hypothesis that more experienced residents would have higher ratings than less experienced residents. Overall reliability was high (α >0.8) with evidence of validity. RPAGE may be an acceptable electronic log of practice data, but may not be acceptable for workplace-based assessment.

Karwowska and Tse in “Keeping busy learners informed: email is most useful for medical residents!” looked for the best method of three to distribute orientation information: email, online, and paper. They found that email is efficient and effective. Resident did not access the wiki site much at all but this may be worth further exploration.

Traboulsi et al. in “Does self-modulated learning vs. algorithm-regulated learning of dermatology morphology affect learning efficiency of medical students?**”** explore whether there might be differences in learning between self-regulated and algorithm-regulated practice. First year medical students at the University of Calgary completed a dermatologic morphology module. The authors randomly assigned them to either a self-regulated arm (at their own discretion students removed cases from the practice pool) or an algorithm-regulated arm (an algorithm determined when the program removed a case). They collected data on mean diagnostic accuracy of the practice sessions and tests and the time spent practicing. Students in the algorithm-regulated arm completed many more cases than those in the self-regulated arm (52.9 vs. 29.3, p<0.001). There were no differences between the two groups of students on the post-test (90% vs. 86%, n = 10) and both groups improved. While the sample was very small and the testing done for short- term retention only, it seems the self-regulated group was more efficient.

Riva and his team of authors in “Medical students’ challenges and suggestions regarding research training: a synthesis of comments from a cross- sectional survey” analyzed comments from 360 medical students who responded to a survey on research opportunities. Among other findings they discovered that students wanted active participation when identifying research opportunities and interested mentors and that there was some uncertainty about research training translating into useable skills. This should give us some pause when pushing students into the research rat race.

Bharwani et al. in “Perceptions of effective leadership in a medical school context” describe how multiple stakeholder groups associated with medical school leadership programs conceptualized the construct of leadership. They conducted 77 semi-structured interviews with six major stakeholder groups: Trainees, Mid-Level University Leaders, Clinician Leaders, Senior University Leaders, Medical Scientists, and Senior Leaders, external to the University. Among other things, they found that participants expected leaders 1) to create a compelling vision and a foster a motivating culture within the organization and 2) to possess integrity, technical competence, communication skills, and a sense of passion about leading.

Rajwani and his team in “Improving the competence and confidence of pulmonary and critical care medicine fellows in performing a cricothyrotomy” describe an evidence-based cricothyrotomy course following the 4-phase lesson plan for simulation. Pulmonary and critical care medicine fellows reported greater confidence (p<0.005) and competence (p<0.002) following this educational intervention. They also showed improvement in knowledge (p<0.003) and skills in two cricothyrotomy techniques. This is a promising module worth trying in other settings.

Pero and Marcotte in “Scaffolding for assessment success: using gradual release of responsibility to support resident transition to competency-based medical education” describe how the General Internal Medicine (GIM) program at Queen’s University uses “scaffolding” in its assessment strategy. The program coordinates early assessments with specific scheduled learning experiences and gradually releases the responsibility for assessment initiation to residents. Other residency training programs could easily implement this approach that is well suited to support Competency by Design.

Elfassy et al. in “Direct-to-consumer advertising of prescription medications: what the Canadian medical trainee needs to know” describe the known risks of direct-to-consumer advertising and what medical students and residents need to know. They end with a call to integrate effective education into existing programs.

We hope you find truthful and useful research in this issue of the CMEJ and that it will help you to avoid both ignorant and malignant actions.
